# One-way helical electromagnetic wave propagation supported by magnetized plasma

**DOI:** 10.1038/srep21461

**Published:** 2016-02-17

**Authors:** Biao Yang, Mark Lawrence, Wenlong Gao, Qinghua Guo, Shuang Zhang

**Affiliations:** 1 School of Physics and Astronomy, University of Birmingham, Birmingham B15 2TT, United Kingdom; 2 State Key Laboratory of Precision Measuring Technology and Instruments, Tianjin University, Tianjin 300072, China; 3 MOE Key Laboratory of Weak-Light Nonlinear Photonics, School of Physics, Nankai University, Tianjin 300071, China

## Abstract

In this paper we reveal the presence of photonic one-way helical surface states in a simple natural system- magnetized plasma. The application of an external magnetic field to a bulk plasma body not only breaks time-reversal-symmetry but also leads to separation of Equi-Frequency Contour surfaces (EFCs) to form topologically nontrivial gaps in k space. Interestingly, these EFCs support topologically protected surface states. We numerically investigate an interface between magnetized plasma, using a realistic model for parameter dispersion, and vacuum, to confirm the existence of one-way scatter-immune helical surface states. Unlike previous proposals for achieving photonic one-way propagation, our scheme does not require the use of artificial structures and should therefore be simple to implement experimentally.

The pursuit of one-way scatter-immune transportation of light has recently become a hot research topic not least because of the clear technological benefits of being able to manipulate electromagnetic waves while maintaining perfect transmission. Its apparent carrier is surface wave, by definition, is a wave bounded by the interface between two semi-infinite media. Traditional surface wave usually endures non-ignorable scattering loss when encountering any interfacial disorders, such as sharp bend. Recently, many kinds of photonic nontrivial surface wave, which show the robust property of one-way scatter-immune transportation, have been proposed by means of mimicking topological electronic system^1^,^2^. The defining feature of topological phases is bulk-edge correspondence. Bulk-edge correspondence, referring to the surprising dependence of boundary excitations on the characteristics of the bulk propagating modes rather than the local properties of the boundary, rebuilds our concepts on energy band theory. After Haldane proposed the photonic analogue of Quantum Hall Effect in 2008^3^,^4^, several significant papers clarifying topological edge states both theoretically^5^,^6^,^7^,^8^,^9^ and experimentally^10^,^11^,^12^,^13^,^14^,^15^ have brought a whole new challenge to traditional optics. Simultaneously, magnetized near-zero-epsilon metamaterials^16^ was also proposed to prove one-way photonic states in 2-D plane. Obviously, a wholly new field-topological photonics^17^ has emerged. However, what cannot be neglected is that fabrication and assembly, especially 3D case for bulk photonic crystal or metamaterials, is still challenging and time consuming. Here, we propose a simple natural system-magnetized plasma to realize helical one-way surface propagation. More importantly, the topological transportation of electromagnetic surface waves can be reconfigurable by simply adjusting the external magnetic field or the plasma density.

Magnetized plasma has been recently investigated to show interesting electromagnetic properties such as sub-diffractional imaging^18^ and magnetic field induced transparency^19^. Under a strong magnetic field, the movement of the free electrons in the cold plasma is confined in the plane perpendicular to the applied magnetic field. On the other hand, the electrons can move freely along the direction of the magnetic field. It has been shown previously that with extremely strong magnetic field, the wave can propagate almost diffractionless along the direction of the magnetic field, in a similar way as wave propagate inside a hyperbolic metamaterials^20^,^21^, which currently is a very active research topic including subwavelength imaging^22^,^23^,^24^, negative refraction^25^, spontaneous emission enhancement with a large Purcell Factor^26^ and even topological ordered metamaterials^9^.

For propagation of electromagnetic wave with angular frequency ω in the lossless plasma, the lossless plasma behaves as a free-electron model, whose electromagnetic response can be described by the Drude permittivity 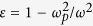, where 
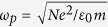
 is the plasma frequency, 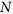 being the electron concentration, e and m denote elementary electron charge and electron mass, respectively. As shown previously^18^, the applied external DC magnetic field leads the isotropic plasma to becoming extremely anisotropic medium because of electrons undergoing cyclotron orbits in the plane perpendicular to the field. If a magnetic field is aligned in the z direction, the electromagnetic response of the lossless magnetized plasma can be described by the following local, homogeneous permittivity tensor^18^,^27^,^28^,


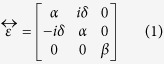


where, 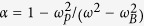, 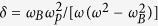 and 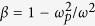. The cyclotron frequency 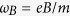 is determined by the applied static magnetic field 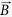.

Considering a wave propagating along + z direction with circular polarizations as base states, this relative permittivity of the magnetized plasma can be reduced to a diagonal tensor 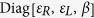, where 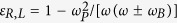, ‘
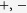
’ correspond to right and left polarized states, respectively. To meet our hyperbolic demand in z direction which is parallel to the static magnetic field, the operating frequency must satisfy 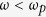 in the lossless condition. Whereas the applied magnetic field draws off-diagonal components into the relative permittivity, the magnetized plasma shows two bandgaps in k-space with condition of 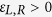. So, the operating angle frequency has to be less than 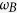 and satisfy 
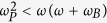
. Based on these constraints, we can find 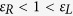, which means EFCs of vacuum is located in the EFCs’ bandgaps of the magnetized plasma, as shown in Fig. 1. In other words, these two materials have overlapped forbidden bands in k space, which is an essential condition to realize one-way scatter-immune transportation. Figure 1 shows corresponding hyperbolic properties and bandgaps of magnetized plasma, it can be seen as the result of a nontrivial transformation from normal hyperbolic metamaterials.

We now turn to studying how these topological features manifest themselves on the boundary of magnetized plasma. In what follows we investigate systems with continuous translational invariance in the z direction, thereby conserving 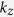. Here, by assuming the surface wave exponentially decays along either direction from y-z plane (i.e., the half space 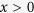 is occupied by isotropic media such as vacuum, whereas the magnetized plasma is located in 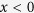), we use the method proposed by Dyakonov in 1988^29^ to briefly calculate these surface wave. In the vacuum side, there are two orthogonal eigen modes which can be expressed as,


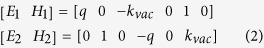


where 
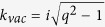
 is imaginary representing decay constant along positive x direction and q is the absolute value of in-plane propagation wave vector. Likewise, we can also write down the decay constant in magnetized plasma side 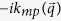 as a function of vector 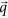. From two independent eigen modes of magnetized plasma it can be obtained through solving Maxwell’s Equations,


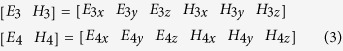


which both still are functions of 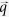. Until now, we have found four eigen modes that are localized on both sides of the surface. What combines them is the electromagnetic boundary condition. Postulating that the tangential components of these fields are continuous at the interface gives us the determinant problem of a 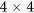 constraint matrix,


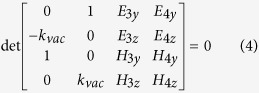


After solving it, in Fig. 2a the results reveal that in the gap of EFCs any given surface can support just one propagating mode, which means that the spatial separation of left and right moving surface waves at certain 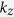 prevents the occurrence of backscattering from any z-invariant disorder as shown in Fig. 2b. Topological order for magnetized plasma is attributed to the presence of a new type of plasmon Weyl points occurring at the plasma frequency of the system^30^.

This immunity to backscattering has also been confirmed using full wave simulations shown in Fig. 3b in which a right moving surface wave propagates seamlessly around a sharp defect. The simulation is performed in the x-y plane for three different propagation constants 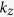 in the shadow area as indicated by ‘A’, ‘B’ and ‘C’ in Fig. 3a. For point ‘A’ and ‘C’, since they are not located in the gap region, the electromagnetic wave can be scattered into the bulk states by z-invariant scatterers. On the other hand, at point ‘B’ where 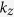 is in the middle of the gap, the surface wave is immune from scattering by the sharp edges, and therefore the numerical simulation confirms that it is a topologically nontrivial surface state. As expected, when the direction of the magnetic field is flipped, the propagation of the surface wave is also switched to the opposite direction. This may enable topological surface states with dynamically reconfigurable properties. Since the collision frequency is several orders of magnitude lower than the operating frequency, this realistic case can still be regard as lossless^18^.

Interestingly, after zooming in the point ‘C’ shown in Fig. 3a where surface state is very close to the EFCs of vacuum, it is discovered that the surface state is not merging into the EFCs of vacuum, rather, it conformally bends around the EFCs of vacuum and goes parallel to the other branch of the EFCs of magnetized plasma. This indicates that even at 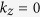 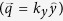, there exists a unidirectional surface state on each surface with dispersion relation expressed as^16^,


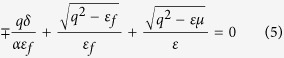


where 
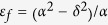
 and 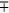 represents to top and bottom case, respectively. What’s important is that this surface mode can only be excited by magnetic current. After substituting the realistic parameters into Eq. 5, we get the mentioned subtle gap between surface state and vacuum bulk state at point ‘C’ is 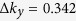. However, since the surface states do not fall in the gap, they are not protected against z invariant defects.

For completeness, we show the polarization states of these nontrivial surface waves in Fig. 4. According to the boundary conditions, the tangential components of electric (E) and magnetic field (H) are continuous across the interface. However, the vertical components of the E and H fields are discontinuous. Specifically, they are related by 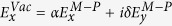. Therefore, the polarization states on the vacuum and the magnetized plasma sides are different. The polarization states on both side of the interface are generally elliptically polarized. Interestingly, the polarization ellipse lies in a plane that is perpendicular to the surface. Another interesting observation is that the polarization ellipse is generally not perpendicular to the Poynting vector. The angle formed between them varies monotonically from 0° for *k*_*z*_ = 0 to 90° for *k* approaching infinity, as shown in Fig. 4b. Moreover, the plot of ellipticity shows that when k approaches infinity, the electric field in vacuum side becomes circularly polarized.

In conclusion, we have theoretically investigated the existence of one-way propagating surface wave between vacuum and magnetized plasma. The simulation confirms the presence of unidirectional backscattering-immune propagation of surface wave based on the simple natural system-magnetized plasma. Although the study has focused on free space magnetized plasma for manipulation of electromagnetic waves at microwave regime, it can be extended to terahertz regime by working with semiconductors with very small effect mass, such as InSb. Thus, this nontrivial edge states may also be observed in THz band by using magnetized semiconductors^31^.

## Additional Information

**How to cite this article**: Yang, B. *et al.* One-way helical electromagnetic wave propagation supported by magnetized plasma. *Sci. Rep.* **6**, 21461; doi: 10.1038/srep21461 (2016).

## Figures and Tables

**Figure 1:**
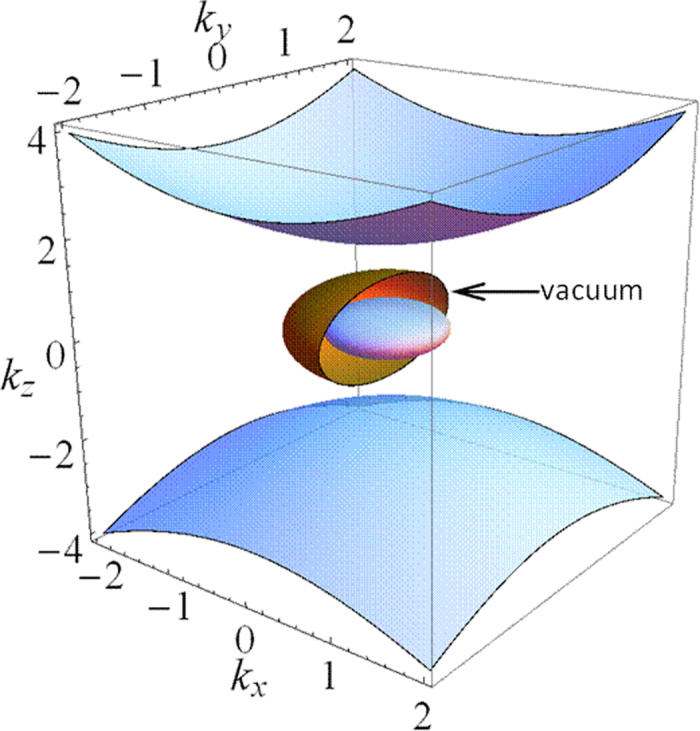
EFCs of magnetized plasma and vacuum. The ordinary mode is wrapped by vacuum (indicated). There is a big gap between the extraordinary mode and vacuum. At the minimal point of upper pseudo hyperbolic branch, light propagates with strict left circular polarization states. However, for 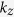 approaching infinity, polarization states become linear. The peak point of upper ordinary semi-sphere shows strict right circular polarization which is opposite to that of the minimal point, because these two states are lifted from a degeneracy point during nontrivial transformation from normal hyperbolic metamaterials. In the negative 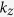, polarization presents opposite properties. In the plane of 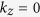, the z component of electric field is zero again, but its polarization is slightly elliptical compared with those pole locations. The permittivity is calculated with respect to 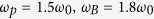. The wave vectors are in the unit of 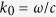.

**Figure 2:**
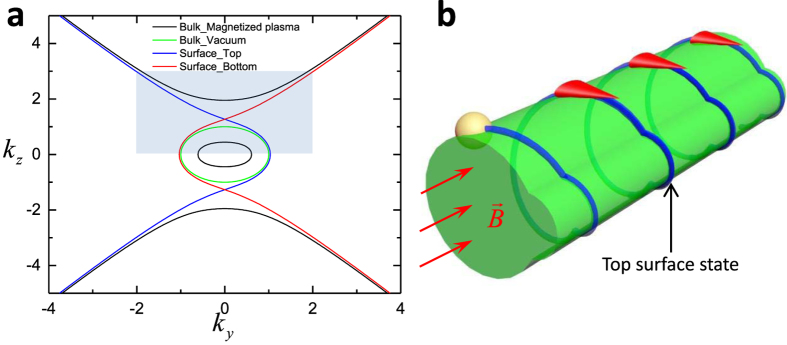
Bulk states and surface states. (**a**) Bulk states and surface states of this system. Top/Bottom refers to the configuration that vacuum is located on the top/bottom of magnetized plasma. (**b**) Schematic view of helical one-way back scattering-immune propagation in 3D. Chiral surface state propagating around the magnetized plasma with added cylindrical shape surrounded by vacuum, despite the existence of raised cylinder back-scattering is forbidden due to the absence of anticlockwise modes.

**Figure 3:**
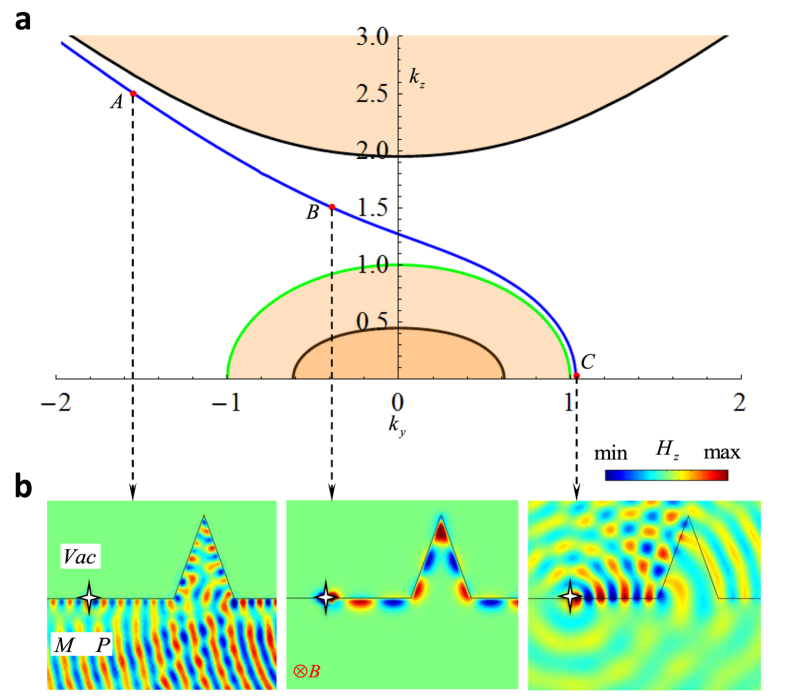
Simulation of topologically protected surface states at the interface between a magnetic plasma and vacuum. (**a**) Magnified shadow area of Fig. 2(a). (**b**) Field distribution simulated by commercial Comsol RF module. The parameter of the magnetized plasma are 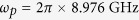, 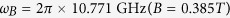 and the operating frequency 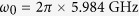.

**Figure 4:**
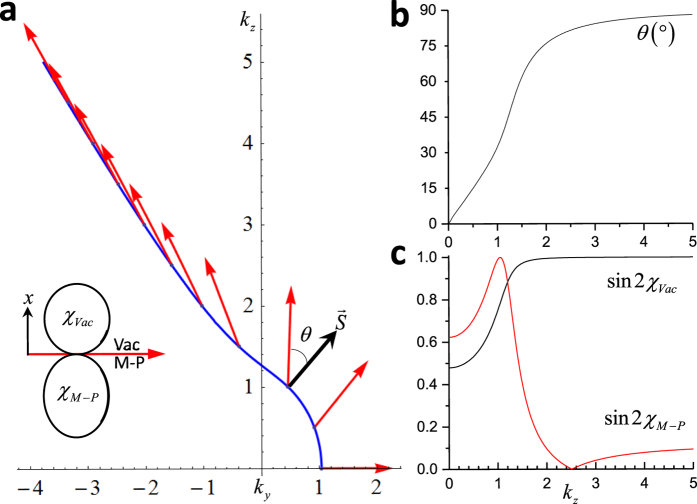
Electric field polarization of the surface states. (**a**) The polarization states of the surface waves in vacuum and in the magnetized plasma. The polarization plane is perpendicular to the interface, therefore from top view, it is projected onto the interface as a linear vector. The insect shows that the polarization states are elliptical. (**b**) and (**c**) Direction of polarization plane and the ellipticities (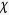) of the polarization states in vacuum and in the magnetized plasma. This figure corresponds to Fig. 3(a) for surface states at 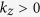.
